# May Measurement Month 2017–2019: A Community-Wide Opportunistic Blood Pressure Screening Campaign in Hong Kong

**DOI:** 10.1155/2021/8891794

**Published:** 2021-01-18

**Authors:** Joyce Tik Sze Li, Amy Shuk Man Lam, Brian Tomlinson, Vivian Wing Yan Lee

**Affiliations:** ^1^Centre for Learning Enhancement and Research, The Chinese University of Hong Kong, 502, Hui Yeung Shing Building, Shatin, Hong Kong; ^2^Department of Medicine & Therapeutics, Faculty of Medicine, The Chinese University of Hong Kong, Shatin, Hong Kong; ^3^Faculty of Medicine, Macau University of Science & Technology, Block P25, Avenida Wai Long, Taipa, Macau

## Abstract

**Introduction:**

Hypertension is a modifiable risk factor for multiple cardiovascular diseases. Early identification and intervention of new cases are crucial to improve patients' outcomes. May Measurement Month (MMM) is an annual global synchronised blood pressure (BP) screening campaign. Participants can have their BP measured at the screening sites. It may be a possible way to identify undiagnosed hypertensive patients in the population.

**Methods:**

It was a cross-sectional study of BP among Hong Kong adults. Multiple screening sites were set in local community pharmacies and on the campus of the Chinese University of Hong Kong. Participants were asked to fill in a questionnaire regarding their demographics, medical history, and social history. Then, they took at least one BP reading using an automated sphygmomanometer after sitting at for 5 minutes. Up to three BP readings were taken and recorded for each participant, with one-minute intervals between readings.

**Results:**

A total of 3224 adults participated in MMM between 2017 and 2019. The average BP among the 3224 participants was 139.8/75.5 mmHg. The prevalence of hypertension was 2282 (70.8%), of which 635 (27.8%) were undiagnosed before MMM. Among the 1647 participants previously diagnosed with hypertension, 1007 (61.1%) had uncontrolled hypertension.

**Conclusion:**

A high number of cases can be identified with untreated, or treated but uncontrolled, hypertension from MMM. Citizens should be encouraged to check BP regularly and take follow-up actions if hypertension is suspected.

## 1. Introduction

Hypertension is a known risk factor for multiple cardiovascular diseases and cerebrovascular events [1, 2]. Raised blood pressure (BP) is the most important modifiable risk factor for premature death [3, 4]. It is estimated that 1.13 billion people worldwide have hypertension, with around two-thirds living in low- and middle-income countries [5]. One of the global targets for non-communicable diseases is to reduce the prevalence of hypertension by 25% by 2025 starting from 2010 [5].

Despite the high number of cases, there are still a lot of the undiagnosed or undertreated patients. The well-known “rule of halves” in hypertension states that roughly only half of all hypertensive cases are diagnosed, half of those diagnosed are treated, and half of those treated are well controlled [6, 7]. Locally, the Population Health Survey (PHS) 2014/15 conducted by the Department of Health revealed that the total prevalence of hypertension among persons aged 15 or above was 27.7% (25.5% for females and 30.1% for males) [8]. Around 47.5% of them were undiagnosed before the PHS. The total prevalence of hypertension increased steadily with age from 4.5% among those aged 15–24 to 64.8% among those aged 65–84.

Hypertension is known as a “silent killer.” While most people with hypertension are unaware of the problem because there may be no warning signs or symptoms, untreated hypertension can lead to serious complications such as coronary heart disease, stroke, heart failure, and renal failure [9–11]. Early identification and intervention of new cases are crucial to minimize preventable death [12, 13]. The Hong Kong Reference Framework for Hypertension Care for Adults in Primary Care Settings has set out recommendations on the prevention and treatment of hypertension among adults [14]. It clearly states the importance of early identification of people with hypertension and recommends BP measurement in all adults from 18 years of age at least every 2 years.

Community-wide BP screening is a possible way to reach out citizens who do not have the habit of having a regular body check up [15, 16]. May Measurement Month (MMM) is an annual global synchronised BP screening campaign led by the International Society of Hypertension (ISH) since May 2017 [17–19]. It aims to raise citizens' awareness on hypertension, collect the scientific evidence needed to help influence global health policy, and make BP screening more widely available around the world through MMM [20]. In 2018, 1,504,963 adults from 89 countries participated in MMM [18]. Hong Kong is one of the MMM participating places. This paper reports the findings of MMM from 2017 to 2019 in Hong Kong.

## 2. Methods

### 2.1. Study Design and Study Population

It was a cross-sectional opportunistic study of BP among adults in Hong Kong. The Chinese University of Hong Kong (CUHK) collaborated with two local chain community pharmacies to set up multiple BP measurement stations. Participants had their BP measured either in the community pharmacy or on the CUHK campus. Pharmacists working in the community pharmacies, as well as staff and students from the Faculty of Medicine, CUHK, helped with BP measurements. Not only did the MMM programs provide an excellent platform for primary care in the community but also for student engagement in community outreach.

Participants were recruited through convenience sampling. Participants aged 18 or above who were able to provide informed consent were recruited. The event was promoted through fliers, posters, press release, and social media. Participants were self-referred to the screening sites.

### 2.2. Procedures

The current study complied with the MMM BP measurement protocol set by the ISH [17–19].

Before BP measurement, the participants were seated with their backs supported and with their legs resting on the ground and in the uncrossed position for 5 minutes. Meanwhile, each participant filled in an anonymous questionnaire regarding their demographics, such as ethnicity, age, height, weight, medical history, social history, and habits of BP monitoring. At least one BP reading was taken using an Omron BP device (Omron Healthcare, Kyoto, Japan), which was an automated sphygmomanometer that measured systolic blood pressure (SBP), diastolic blood pressure (DBP), and heart rate. Upon mutual agreement with participants, three BP readings were taken and recorded, with one-minute interval between readings. In the current project, hypertension was defined as a systolic BP ≥140 mmHg, diastolic BP ≥90 mmHg, or on at least one antihypertensive medication taken for raised BP. Data collected were analysed centrally by the MMM project team.

Dietary and lifestyle information would be provided to participants found to be hypertensive. They were advised to reduce salt intake, reduce alcohol intake, engage in regular physical exercise for at least 30 minutes on most of the days of the week, have at least 5 portions of fruit and vegetables per day, and reduce weight aiming for a body mass index (BMI) target of <25 kg/m^2^. They were also advised to have home BP monitoring.

## 3. Results

A total of 3224 adults participated in MMM between 2017 and 2019. Since the questionnaire differed slightly between years and the data collection was incomplete for some of the items, numbers used in different analyses varied. Participant demographics are shown in Table 1. Among the 3224 participants, 22.3% were male and 77.7% were female. Their mean age was 71.9 ± 13.0. There were 17.7% of participants with diabetes, 9.9% with history of heart attack, and 4.6% with history of stroke. As for social history, 2.8% of participants reported tobacco use and 5.4% reported alcohol consumption.

In total, 308 (9.6%) participants took BP readings three times, 9 participants (0.3%) took BP readings twice, and 2907 (90.2%) took BP reading once. Of the 308 participants who took three readings, the first reading was the highest and the third reading was the lowest. Mean values of the first and third reading were 125.9/78.6 mmHg and 122.3/76.9 mmHg, respectively.

Table 2 showed the mean BP of participants with regard to their first readings. When considering the first BP readings among the 3224 participants, their average BP was 139.8/75.5 mmHg. There were 1647 out of 3224 participants (51.1%) who had been previously diagnosed with hypertension. Among the 1543 participants who had not been diagnosed with hypertension, 635 (41.2%) of them were found to be hypertensive. The total number of participants with hypertension was 2282 (70.8%). Among the 1647 participants previously diagnosed with hypertension, 1007 (61.1%) had uncontrolled hypertension.

Tables 3 and 4 showed the mean BP of participants stratified according to medical history and social history. The mean BP of participants with diabetes, history of heart attack, and history of stroke was 141.7/73.1 mmHg, 140.4/72.7 mmHg, and 143.7/74.4 mmHg, respectively. The proportion of participants with hypertension was 53.2%, 51.1%, and 59.7%, respectively. The mean BP of participants who used tobacco and consumed alcohol was 130.7/76.6 mmHg and 140.2/79.3 mmHg, respectively. The proportion of participants with hypertension was 36.8% and 51.8%, respectively.

## 4. Discussion

May Measurement Month is a community-wide opportunistic hypertension screening programme. Participants volunteer to have their BP measured at the screening sites. The results were based on one single set of readings. Numerous factors such as noise from the surrounding, exercise, nicotine or caffeine intake prior to BP measurement, and white coat hypertension could affect the accuracy of measurements [21–23]. Thus, participants were informed that the results should not be treated as a diagnosis. In order to increase the reliability of BP measurements, MMM suggested all participants to take three readings and calculate the mean of the second and third reading for interpretation. The MMM BP measurement protocol was similar to that recommended by the World Health Organisation (WHO) [24]. Nevertheless, it was not practical in some of the screening sites in Hong Kong due to multiple reasons, such as lack of space for participants to sit and rest in the community pharmacies, inadequate manpower in the pharmacy counters, and participants had hectic schedules and were not eager to take three readings. Consequently, data interpretation was mainly based on the first readings.

Combining the results form MMM17, MMM18, and MMM19, 70.8% of participants were found to be hypertensive, of which 27.8% were undiagnosed before MMM. This number was comparable to that reported in PHS 2014/15 [8]. PHS 2014/15 revealed that, among the 27.7% of citizens aged 15 or above found to have hypertension, 47.7% of them were undiagnosed before PHS. In the subgroup of citizens aged 65–84, 64.8% were found to have hypertension and 20.9% of them were undiagnosed before PHS. The mean age of MMM participants was around 72 years, and the results were similar to that found in the subgroup of PHS participants aged 65–84. Therefore, although MMM provided BP screening service for all adults, the findings between 2017 and 2019 might be more representative for the older population.

Among the 1,508,130 participants in MMM19 worldwide, 34.9% were found to have hypertension [19]. There were 18.6% of participants who were not taking antihypertensive medications found to have hypertension. Among those already taking antihypertensive medications, 38.3% had uncontrolled hypertension. Among the 280,863 participants from East Asia, 30.6% had hypertension. There were 14.8% of participants who were not taking antihypertensive medications found to have hypertension and 33.4% of participants taking antihypertensive found to have uncontrolled hypertension.

The proportion of Hong Kong participants with hypertension appeared to be higher than the average results worldwide, as well as in East Asia. Nevertheless, the numbers presented by the MMM project team were analysed using the mean BP from the second and third readings. The proportions were also calculated after imputation and standardization for age and sex according to the WHO world-standard population. Direct comparison between numbers was not feasible. However, it was clear that the proportion of participants newly found to have hypertension was high, which indicated that community-wide screening might be useful to identify new cases. It was essential for participants to take follow-up actions so as to confirm the diagnosis and receive nonpharmacological or pharmacological interventions when indicated. The risk of cardiovascular diseases could only be reduced if participants were motivated and willing to take further actions.

Participants in MMM were probably aware of the importance of BP monitoring. In 2018, there were 187 out of 192 (97.4%) participants who had measured BP before MMM. Among them, 149 out of 187 (79.7%) had measured BP within the previous 12 months. It was understandable as those who volunteered to participate were expected to be more attentive to own health. Participants also appeared to have healthier lifestyle as compared to the whole Hong Kong population. There were 2.8% of participants who smoked and 5.4% who consumed alcohol. These proportions were lower than reported in Health Behaviour Survey 2018/19, which stated that 17.9% of the population aged 15 or above had even smoked and 8.8% of the population drank alcohol at least once a week [25]. Nevertheless, the proportion of participants with uncontrolled hypertension was high. Among those previously diagnosed with hypertension, 61.1% had uncontrolled hypertension. MMM could be a platform to advocate ambulatory BP monitoring and encourage participants to measure BP regularly.

The purpose of screening is to promote individual health through early detection of diseases [26]. It is particularly important if disease prognosis can be improved significantly by early treatment [27]. However, mass screening can be costly, especially when the equipment needed is expensive or a specific technique is needed during the collection process [27]. The screening should also be tailored to the characteristics of the population [28]. Thus, it is essential to select targeted diseases and screening tests for mass screening. On the other hand, community health screening would be less effective if the participation rate is low. Unlike schoolchildren who may have health check during periodic medical visits, screening among adults is often opportunistic [26]. Previous studies have identified the barriers and facilitators to health screening among adults [29]. Some common barriers include fear of getting disease and consequence, low risk perception, painful and uncomfortable screening procedure, lack of time, and embarrassing screening procedure [30–33]. From the experience in MMM, BP screening was easy to perform. The automated sphygmomanometers were reusable, so the equipment cost was low. The measurement processes were quick, and no uncomfortable or embarrassing procedures were involved. Participants acceptance would be higher. Yet, it successfully identified new hypertension cases, which might reduce the risk of complications and comorbidities. Therefore, we believe community-wide BP screening should be encouraged.

Advancement in technology has made ambulatory and continuous health monitoring feasible. Consumer-grade digital devices such as smart watches and mobile applications allow self-monitoring of personal activity [34]. They can provide feedbacks and analyses to users based on activity measures. Data captured can facilitate communication with healthcare providers and family members. These devices can benefit treatment and research in multiple areas, such as seizure detection, heart rate monitoring, diabetes self-management, and detection of emotional states [35–38]. With the help of smart watches, citizens can have their BP measured at rest, during daily activities, and during exercise. As the number of smart watch users grows, the amount of data collected is expected to increase as well. It may be an alternative means of screening. Still, postdiagnosis intervention is essential to promote patients' outcome. Users should be encouraged to adopt lifestyle modification and seek medical attention if persistently elevated BP is detected. It will require interprofessional collaboration to develop a system that provides holistic care to citizens, including BP measurement, postscreening counselling, and data processing.

A major aim of MMM is to raise people's awareness on hypertension [20]. The screenings were convenient, timely, and free of charge which attracted volunteers to participate. PHS 2014/15 reported that 41.2% of citizens aged 65–74 had the habit of regular medical checkup [8]. While regular body checkup is essential, frequent self-monitoring of health parameters could also improve disease control [39–43]. Due to the COVID-19 pandemic, a lot of the community health screening activities planned in 2020 have been cancelled, including MMM20. Community centres are temporarily closed; thus, members are unable to have regular BP measurement there. While it is hoped that health screening can be resumed as soon as the outbreak dies down, patient empowerment is also critical. It is crucial to educate and provide support to citizens so that they can self-monitor BP. Besides, lifestyle changes during the COVID-19 outbreak such as reduced physical activities, reduced exercise level, and increased consumption of preserved food can hamper patients' BP control [44–47]. It is essential for citizens to recognise the long-term impact of raised BP and increase control over their own health.

The present study has a few limitations. Firstly, the number of participants in MMM17, MMM18, and MMM19 were different. Factors such as promotion strategies, number and location of screening sites, and number of helpers recruited might have affected the scale of event. In addition, due to the social movement in 2019, some of the screening sites were closed. The number of participants in MMM19 was greatly reduced. Thus, it was hard to compare the results across years. Secondly, the proportion of female participants was higher than that of male, which implied a possibility of selection bias. This phenomenon correlated with the pattern observed in other public screening or health outreach activities in Hong Kong [48–50]. Some previous studies also revealed gender differences in primary care consultation rates [51, 52]. It was suggested that female citizens might be more motivated to participate in health screening activities. Thirdly, the sample size in some of the subgroups was small, which makes the results less representative. Some participants did not complete the whole questionnaire, and we could not assign them to any subgroups. Also, the questionnaire was modified each year and some questions were newly added. Therefore, the number of responders in those questions was fewer.

## 5. Conclusions

The results from MMM reveal that high numbers of people can be identified with untreated, or treated but uncontrolled, hypertension from an opportunistic screening campaign. Health screening should be hosted regularly to identify new cases and raise citizens' awareness on hypertension. Citizens should be encouraged to self-monitor BP and take follow-up actions if hypertension is suspected.

## Figures and Tables

**Table 1:** Participant demographics.

	2017	2018	2019	Total
No of participants	2358	717	149	3224
Mean age	76.1 ± 8.2	60.3 ± 16.0	60.2 ± 17.2	71.9 ± 13.0
Mean BMI	—	23.3 ± 4.0	23.4 ± 5.5	—

Gender				
Male	488/2358 (20.7%)	184/717 (25.7%)	47/149 (31.5%)	719/3224 (22.3%)
Female	1870/2358 (79.3%)	533/717 (74.3%)	102/149 (68.5%)	2505/3224 (77.7%)
Ethnicity				
East Asian	—	187 (93.0%)	143 (96.0%)	—
South-East Asian		12 (6.0%)	4 (2.7%)	
White		2 (1.0%)	1 (0.7%)	
South Asian		0 (0.0%)	1 (0.7%)	

Medical history				
Hypertension	1313/2358 (55.7%)	277/683 (40.6%)	57/149 (38.3%)	1647/3190 (51.6%)
On antihypertensive medications	—	55/201 (27.4%)	56/149 (37.6%)	—
Diabetes	475/2358 (20.1%)	84/717 (11.7%)	12/149 (8.1%)	571/3224 (17.7%)
Previous heart attack	289/2358 (12.3%)	16/627 (2.6%)	4/149 (2.7%)	309/3134 (9.9%)
Previous stroke	124/2358 (5.3%)	17/627 (2.7%)	3/149 (2.0%)	144/3134 (4.6%)

Social history				
Tobacco use	48/2358 (2.0%)	30/631 (4.8%)	9/141 (6.4%)	87/3130 (2.8%)
Alcohol consumption	107/2358 (4.5%)	47/628 (7.5%)	16/149 (10.7%)	170/3135 (5.4%)

**Table 2:** Mean BP of participants using the first reading.

	2017	2018	2019	Total
Mean SBP	142.4	134.6	126.7	139.8
Mean DBP	74.4	78.8	77.5	75.5
Number with hypertension	1319/2358 (55.9%)	292/717 (40.7%)	44/149 (29.5%)	1655/3224 (51.3%)
Newly found to have hypertension	510/1823 (28.0%)	103/380 (27.1%)	22/79 (27.8%)	635/2282 (27.8%)
Uncontrolled BP with previous diagnosis of hypertension	809/1313 (61.6%)	176/277 (63.5%)	22/57 (38.6%)	1007/1647 (61.1%)

**Table 3:** Mean BP of participants according to medical history.

Year	Medical history	SBP	DBP	Number with hypertension	Proportion with hypertension
2017	Diabetes	141.9	72.4	256/475	53.9
	Previous heart attack	139.9	72.1	144/289	49.8
	Previous stroke	144.3	74.1	78/124	62.9

2018	Diabetes	142.4	77.2	43/84	51.2
	Previous heart attack	144.3	81.8	10/16	62.5
	Previous stroke	141.6	77.2	8/17	47.1

2019	Diabetes	129.3	72.7	5/12	41.7
	Previous heart attack	158.5	78.5	4/4	100
	Previous stroke	128.0	72.7	0/3	0

Total	Diabetes	141.7	73.1	304/571	53.2
	Previous heart attack	140.4	72.7	158/309	51.1
	Previous stroke	143.7	74.4	86/144	59.7

**Table 4:** Mean BP of participants according to social history.

Year	Social history	SBP	DBP	Number with hypertension	Proportion with hypertension (%)
2017	Tobacco use	135.8	75.7	21/48	43.8
	Alcohol consumption	142.6	77.1	60/107	56.1

2018	Tobacco use	124.1	77.1	9/30	30.0
	Alcohol consumption	138.0	83.7	22/47	46.8

2019	Tobacco use	126.0	79.2	2/9	22.2
	Alcohol consumption	130.8	81.4	6/16	37.5

Total	Tobacco use	130.7	76.6	32/87	36.8
	Alcohol consumption	140.2	79.3	88/170	51.8

## Data Availability

The datasets used and analysed during the current study are available from the corresponding author on reasonable request.

## Abbreviations

BP: Blood pressure
CUHK: The Chinese University of Hong Kong
DBP: Diastolic blood pressure
MMM: May Measurement Month
PHS: Population health survey
SBP: Systolic blood pressure
WHO: World Health Organisation.
